# Updated adolescent diagnostic criteria for polycystic ovary syndrome: impact on prevalence and longitudinal body mass index trajectories from birth to adulthood

**DOI:** 10.1186/s12916-020-01861-x

**Published:** 2020-12-11

**Authors:** Chau Thien Tay, Roger J. Hart, Martha Hickey, Lisa J. Moran, Arul Earnest, Dorota A. Doherty, Helena J. Teede, Anju E. Joham

**Affiliations:** 1 Monash Centre for Health Research and Implementation, School of Public Health and Preventive Medicine, Monash University, Clayton, Victoria Australia; 2 Departments of Endocrinology and Diabetes, Monash Health, Clayton, Victoria Australia; 3 Division of Obstetrics and Gynaecology, Faculty of Health and Medical Sciences, The University of Western Australia, Perth, Western Australia Australia; 4 Department of Obstetrics and Gynaecology, University of Melbourne and The Royal Women’s Hospital, Melbourne, Victoria Australia

**Keywords:** Polycystic ovary syndrome, Adolescent, Diagnosis, Weight, Body mass index, Longitudinal

## Abstract

**Background:**

Polycystic ovary syndrome (PCOS) is challenging to diagnose. While the 2003 Rotterdam criteria are widely used for adults, the 2018 international PCOS guideline recommended updated Rotterdam criteria with both hyperandrogenism and oligo-anovulation for adolescents based on evidence-informed expert consensus. This study compared the prevalence of PCOS using updated and original Rotterdam criteria in community-based adolescents and explored long-term body mass index (BMI) trajectories across different diagnostic phenotypes.

**Methods:**

Overall, 227 postmenarchal adolescent females from the prospective cohort Raine Study undertook comprehensive PCOS assessment at age 14–16 years. Detailed anthropometric measurements were collected from birth until age 22 years. Cross-sectional and longitudinal BMI were analyzed using *t* tests and generalized estimating equations.

**Results:**

PCOS was diagnosed in 66 (29.1%) participants using original criteria versus 37 (16.3%) participants using updated Rotterdam criteria. Using updated criteria, participants with PCOS had higher BMI than participants without PCOS from prepubertal. Only the phenotype meeting the updated criteria was significantly associated with higher long-term BMI gain whereas other PCOS phenotypes had similar BMI trajectories to participants without PCOS (*p* < 0.001).

**Conclusions:**

The use of the 2018 updated Rotterdam criteria reduces over-diagnosis of PCOS in adolescents and identifies those at the greatest risk of long-term weight gain, a key contributor to disease severity and long-term health implications. The BMI trajectories of females with PCOS on updated criteria diverge prepubertally compared to those without PCOS. This work supports targeting adolescents diagnosed with PCOS on the 2018 updated criteria for early lifestyle interventions to prevent long-term health complications.

## Background

Polycystic ovary syndrome (PCOS) is the most common endocrinopathy in women of reproductive age with an estimated prevalence of 8–13% [1]. Its pathogenesis includes insulin resistance and hyperandrogenism which drive the reproductive (menstrual dysfunction, infertility), metabolic (metabolic syndrome, diabetes, cardiovascular risk factors), and psychological (anxiety, depression, low quality of life) complications [2]. Given the high prevalence and diverse features across the lifespan, as well as the high prevalence of obesity which further exacerbates its clinical features, PCOS contributes to the global burden of disease [3]. It is therefore imperative to recognize the condition early to facilitate interventions and prevent complications.

Being a heterogeneous disorder, the diagnosis of PCOS is difficult and often delayed [4]. PCOS diagnosis is based on oligo-anovulation (OA), biochemical or clinical hyperandrogenism (HA), and polycystic ovary morphology (PCOM) on ultrasound extending across the original 1990 National Institutes of Health (NIH) criteria (OA and HA) [5], the 2003 Rotterdam criteria (any two of OA, HA, and PCOM) [6], and the Androgen Excess and Polycystic Ovary Syndrome (AE-PCOS) Society criteria (HA and OA or PCOM or both) [7]. The Rotterdam criteria are now widely accepted and generate four possible diagnostic PCOS phenotypes in adult women: (A) OA + HA + PCOM, (B) OA + HA, (C) HA + PCOM, and (D) OA + PCOM [6]. The Rotterdam criteria are recommended and endorsed by the 2018 international PCOS evidence-based guideline, which was co-developed based on unprecedented evidence synthesis and best practice methods, by world-leading multidisciplinary clinicians and researchers across 37 societies from 71 countries, with consumer engagement [8].

PCOS is more challenging to diagnose in adolescents, as menstrual irregularity and multi-follicular ovaries are part of normal pubertal physiology and the application of adult criteria results in a high prevalence and may over-diagnose PCOS [9, 10]. Available recommendations on adolescent PCOS diagnostic criteria are inconsistent. The 2018 international PCOS guideline updated the Rotterdam criteria and now recommends applying OA and HA while avoiding PCOM for PCOS diagnosis in adolescents [8]. However, this evidence-informed recommendation was ultimately based on expert consensus with limited evidence on the most accurate diagnostic approach in adolescents and on the natural history of PCOS phenotypes over time. It remains unclear if the 2018 updated Rotterdam criteria capture adolescents with PCOS who are at the greatest risk of long-term complications and would benefit the most from lifestyle preventative interventions [8].

Long-term weight gain is a major health concern for women with PCOS and a key pathophysiological contributor to PCOS severity [4]. More than 60% of women with PCOS are above healthy body mass index (BMI), exacerbating metabolic, reproductive, and psychological features of PCOS [11, 12]. These effects can be ameliorated by 5–10% weight loss, and lifestyle intervention to prevent weight gain and promote weight loss is therefore the cornerstone of PCOS management [8, 13]. However, existing studies examining the natural history of weight gain in women with PCOS are limited to largely clinic-based adult populations and women with self-reported PCOS and do not differentiate across various PCOS diagnostic criteria or phenotypes [14–22].

To address research priorities and evidence gaps, the aims of the present study in an unselected adolescent population were threefold. Firstly, we aimed to examine the impact of the original 2003 versus the 2018 updated Rotterdam criteria on the prevalence of PCOS diagnosis. Secondly, we aimed to examine the natural history of BMI trajectories in women with and without PCOS from birth until young adulthood, applying both the original and updated adolescent Rotterdam criteria. Thirdly, we aimed to determine BMI trajectories across adolescent phenotypes.

## Methods

### Study design and setting

The Raine Study is a prospective cohort study aiming to investigate the influences of familial, intrauterine, perinatal, and environmental factors on health across the lifespan [23]. Pregnant women between 16 and 20 weeks of gestation who attended public and private antenatal clinics in Western Australia were recruited from 1989 to 1991. More than 2900 pregnant women enrolled in the study and resulted in 2868 live births [23]. To date, the cohort has been followed up for more than 20 years with greater than 70% of the participants still engaged in the study [23]. Data were collected from four generations (mothers and partners originally recruited into the study (Gen1), Raine Study participants (Gen2), offspring of the participants (Gen3), and grandparents of the participants (Gen0)) in the form of surveys, physical examination, and clinical laboratory testing. Between ages 14 and 16, 723 postmenarchal Gen2 adolescent females were invited to participate in the Menstruation in Teenagers Study which involved the collection of self-reported menstrual diary, urinary progesterone analysis, clinical assessment of hirsutism and acne, biochemical measurement of androgen profile, and ultrasound evaluation of ovarian follicles [24]. A total of 244 Gen2 females consented to participate, and their mean age at assessment was 15.2 years [9, 24]. All follow-up assessments were approved by the ethics committees of King Edward Memorial Hospital and/or Princess Margaret Hospital. Further details of the Raine Study are available at www.rainestudy.org.au.

### Outcomes

The primary outcomes of this study were PCOS prevalence and BMI calculated as weight in kilograms per meter squared of height at each follow-up assessment. Participants’ anthropometric data were measured at birth and at ages 1, 2, 3, 5, 8, 10, 14, 16, 20, and 22 by trained research assistants using standardized protocols [25]. Anthropometric data collection was limited to 600 participants of the entire cohort at age 2 due to limitations in funding [25]. Length or height was measured using the Harpenden Neonatometer to the nearest 0.1 cm by two people at birth and age 1 in a supine position [25]. From age 2 onwards, height was measured using a Holtain stadiometer in an anatomical position with shoes off and heels, bottom, and head against a board [25]. Weight was measured with light clothing (running shorts and singlet top) to the nearest 100 g using calibrated hospital scales at birth and Wedderburn digital chair scales from age 1 onwards [25].

### Exposure

The primary exposure was PCOS with the diagnosis ascertained using the original Rotterdam criteria (two out of three clinical features) and updated Rotterdam criteria (OA and HA) (see Table 1 for phenotypes). OA was assessed by a combination of menstrual diary and 12-weekly urinary progesterone metabolite PdG analyses [9, 24]. OA was defined as menstrual cycle length less than 21 or more than 35 days (as the time of assessment was approximately 3 years postmenarche), or where the cycle length varied by more than 4 days, or the urinary PdG to creatinine ratio was less than three times baseline secretion in at least two of the months assessed [9, 24]. Secondary causes of menstrual irregularity such as thyroid disorders and hyperprolactinemia were excluded in all participants [9, 26]. Androgen profile was measured during the early follicular phase (days 2 to 6 of the menstrual cycle) between 15:30 and 16:30 to account for the diurnal variation of androgen production and to fit in with the participants’ school commitments [9, 24]. Total testosterone was measured using a double-antibody radioimmunoassay (DSL-4100, Beckman, Australia: lower limit of sensitivity 0.347 nmol/L; conversion factor to conventional units divide by 0.347 for nanograms per deciliter, intraassay and interpatient coefficients of variation are 6% and 15% at the 1 nmol/L concentration, respectively); sex hormone-binding globulin (SHBG) was measured using a non-competitive liquid-phase immunoradiometric assay (SHBG-IRMA kit; Orion Diagnostica, Espoo, Finland: lower limit of sensitivity 1.3 nmol/L, interassay and intrapatient coefficients of variation 2.0 to 8.6% and 15.4%, respectively) [9, 24, 26]. Biochemical HA was defined as the top 25th centile for circulating free testosterone concentrations (calculated using the Vermeulen equation based on total testosterone and SHBG concentrations, conversion factor to conventional units divide by 0.347 for picograms per deciliter) which was at least 24.45 pmol/L for this data set [9, 24, 26]. A trained nurse evaluated clinical HA using the modified Ferriman-Gallwey score of ≥ 8 to determine hirsutism [9, 24]. PCOM was determined using adult criteria (defined as ≥ 1 ovary ≥ 10cm^3^ in volume or ≥ 12 follicles between 2 and 9 mm diameter) [27] and evaluated using transabdominal ultrasound with a full bladder during the early follicular phase [9, 24, 26, 28]. All ultrasounds were performed by one of two experienced gynecological ultrasonographers while the images were evaluated by one expert radiologist. Either a 5–2-MHz transducer (U22; Philips Medical Systems, Bothell, WA) or a 4-MHz transducer (Voluson 730 Expert; General Electric Milwaukee, WI) was used. The uterine and ovarian volumes were estimated using the formula 0.523 × length × width × height of the organ [29, 30]. Antral follicles were defined as follicles < 10 mm in diameter. Follicular number was assessed by scanning each ovary from the inner margin to the other margin in a longitudinal cross-sectional scanning plane. If a follicle ≥ 10 mm was seen, the ultrasound was repeated in the early follicular phase of the next cycle [9, 24, 26, 28].

**Table 1:** PCOS phenotypes of each comparison group

Group	OA + HA + PCOM	OA + HA	HA + PCOM	OA + PCOM	OA only	HA only	PCOM only	No features
Original PCOS (*n* = 66)	✓	✓	✓	✓				
Updated PCOS (*n* = 37)	✓	✓						
Original non-PCOS (*n* = 161)					✓	✓	✓	✓
Updated non-PCOS (*n* = 190)			✓	✓	✓	✓	✓	✓

*HA* hyperandrogenism, *OA* oligo-anovulation, *PCOM* polycystic ovary morphology, *PCOS* polycystic ovary syndrome

### Covariates

Maternal antenatal information was collected by researchers from maternal medical records. Age at menarche, education level, employment status, smoking status, relationship status, family income, and personal income were collected from surveys at multiple follow-up points. Physical activity was assessed using the International Physical Activity Questionnaire (IPAQ) [31] during age 20 and 22 follow-ups, and the subsequent metablic equivalent (MET)-minutes per week were computed. Energy intake (kcal) was assessed at age 14 via 3-day food diaries with clarification through a follow-up phone call by a dietitian [32].

### Statistical analysis

Analyses were restricted to Gen2 participants in the Menstruation in Teenagers Study who were not on combined oral contraceptive pills and who had a complete assessment of PCOS features. Participants’ characteristics and anthropometry were cross-tabulated using means and standard deviation (SD) or median and interquartile range (IQR) for continuous variables and frequencies for categorical variables. Differences between PCOS status subgroups were assessed using independent *t* test, Fisher’s exact test, Pearson *χ*^2^ test, or Mann-Whitney test as appropriate. Cross-sectional analysis of group-level BMI by PCOS status at each follow-up time point was summarized using means and SD and compared using independent *t* tests. Longitudinal analysis of BMI was performed using generalized estimating equations (GEE) (Gaussian family, identity link, exchangeable covariance structure) which account for between-subjects and within-subjects’ relationships, as well as incomplete follow-up data. BMI change over time was assessed by including PCOS status by time or PCOS phenotypes (non-PCOS, OA + HA, HA + PCOM, OA + PCOM) by time as interaction terms. Covariates were included in the multivariable GEE model if they exhibited a *p* value of < 0.1 in the univariate model. The final included covariates were age of menarche, family income at age 14, and smoking and marital status at age 22. BMI trajectories were compared between participants with and without PCOS by their PCOS phenotypes. Stata software version 15 (StataCorp, College Station, TX) was used for statistical analysis.

## Results

### Participants characteristics and prevalence of PCOS

Of the 244 participants, 17 were excluded due to oral contraceptive pill usage (*n* = 12) and incomplete PCOS assessment (*n* = 5). The remaining 227 were included in our analysis, and the dataset contained a total of 1909 anthropometric measurements allowing BMI calculation from birth until age 22. The median number of anthropometric measurements per participant was 9 (range 4–10).

PCOS was diagnosed in 66 participants (prevalence of 29.1%, 95% confidence interval (CI) 23.5–35.4%) using the original Rotterdam criteria and 37 participants (prevalence of 16.3%, 95% CI 12.0–21.7%) using the 2018 updated Rotterdam criteria (Table 2, Table 1). Participants with and without PCOS on both diagnostic criteria had similar antenatal history, gestational age at delivery, birth measurements, age of menarche, family income, personal income, relationship status, and smoking status. While daily energy intake at age 14 appeared similar for participants with and without PCOS on either diagnostic criteria, participants with PCOS based on the original Rotterdam criteria were less physically active than participants without PCOS (Table 2). The prevalence of participants in each BMI category at age 22 among participants with and without PCOS was similar using the original Rotterdam criteria but different among the subgroups in the 2018 updated adolescent Rotterdam criteria (Table 2).

**Table 2:** Participants’ characteristics

	Overall, *n* = 227	Original 2003 Rotterdam criteria			Updated 2018 adolescent Rotterdam criteria		
		Original non-PCOS, *n* = 161 (70.93%)	Original PCOS, *n* = 66 (29.07%)	*p* value	Updated non-PCOS, *n* = 190 (83.70%)	Updated PCOS, *n* = 37 (16.30%)	*p* value
Diagnostic criterion, *n* (%)							
OA	117 (51.54)	60 (37.27)	57 (86.36)	< 0.001	80 (42.11)	37 (100.00)	< 0.001
HA	60 (28.30)	14 (9.40)	46 (73.02)	< 0.001	23 (13.14)	37 (100.00)	< 0.001
PCOM	78 (35.29)	29 (18.35)	49 (77.78)	< 0.001	58 (31.02)	20 (58.82)	< 0.001
Free testosterone level (mean ± SD)	20.61 ± 13.33	16.42 ± 7.65	30.37 ± 17.94	< 0.001	17.00 ± 8.76	37.77 ± 17.52	< 0.001
Ferriman-Gallwey score (median, IQR)	0 (0, 2)	0 (0, 2)	0 (0, 2)	0.863	0 (0, 2)	0 (0, 4)	0.7352
Age of menarche (mean ± SD)	12.38 ± 1.09	12.45 ± 1.08	12.21 ± 1.12	0.131	12.44 ± 1.07	12.11 ± 1.17	0.094
**Anthropometric characteristics**							
Birth measurements (mean ± SD)							
Birth length (cm)	48.54 ± 2.85	48.51 ± 2.87	48.62 ± 2.83	0.801	48.58 ± 2.79	48.31 ± 3.19	0.592
Birth weight (g)	3281.17 ± 600.40	3287.24 ± 590.66	3266.36 ± 627.90	0.813	3293.06 ± 586.96	3220.14 ± 670.58	0.500
Birth head circumference (cm)	34.09 ± 1.87	34.13 ± 1.83	34.00 ± 1.97	0.631	34.15 ± 1.79	33.76 ± 2.26	0.243
BMI categories at age 22, *n* (%)				0.066			0.003
Underweight/healthy (< 25 kg/m^2^)	113 (49.78)	88 (54.66)	25 (37.88)		104 (54.75)	9 (24.32)	
Overweight (≥ 25 to ≤ 29.9 kg/m^2^)	33 (14.54)	22 (13.66)	11 (16.67)		26 (13.68)	7 (18.92)	
Obese (≥ 30 kg/m^2^)	81 (35.68)	51 (31.68)	30 (45.45)		60 (31.58)	21 (56.76)	
**Demographic characteristics**							
Annual family income at age 14, *n* (%)				0.767			0.886
< $16,000 AUD	8 (3.64)	7 (4.55)	1 (1.52)		7 (3.83)	1 (2.70)	
$16,000–40,000 AUD	52 (23.64)	37 (24.03)	15 (22.73)		43 (23.50)	9 (24.32)	
$40,000–78,000 AUD	83 (37.73)	56 (36.36)	27 (40.91)		67 (36.61)	16 (43.34)	
> $78,000 AUD	77 (35.00)	54 (35.06)	23 (34.85)		66 (36.07)	11 (29.73)	
Personal weekly income at age 22, *n* (%)				0.071			0.191
< $116 AUD	17 (10.56)	12 (9.84)	5 (12.82)		15 (10.49)	2 (11.11)	
$116–604 AUD	83 (51.55)	69 (56.56)	14 (35.90)		77 (53.85)	6 (33.33)	
> $604 AUD	61 (37.89)	41 (33.61)	20 (51.28)		51 (35.66)	10 (55.56)	
Education level at age 22, *n* (%)				0.169			0.032
≤ year 12	92 (53.80)	70 (53.85)	22 (53.66)		80 (52.98)	12 (60.00)	
College/others	35 (20.47)	23 (17.69)	12 (29.27)		28 (18.54)	7 (35.00)	
University or above	44 (25.73)	37 (28.46)	7 (17.07)		43 (24.48)	1 (5.00)	
Employed at age 22, *n* (%)	155 (87.57)	115 (88.46)	40 (85.11)	0.550	134 (88.16)	21 (84.00)	0.559
Relationship status at age 22, *n* (%)				0.717			0.781
Single	61 (35.67)	47 (36.15)	14 (34.15)		52 (34.67)	9 (42.86)	
In a relationship	106 (61.99)	79 (60.77)	27 (65.85)		94 (62.67)	12 (57.14)	
Married	4 (2.34)	4 (3.08)	0 (0)		4 (2.67)	0	
**Lifestyle characteristics**							
Smoking at age 22, *n* (%)	21 (11.80)	12 (9.23)	9 (18.75)	0.114	17 (11.11)	4 (16.00)	0.504
Physical activity, median (IQR)							
Age 20 (metabolic minutes/week)	1989 (844, 3960)	2142 (1050, 4158)	1492 (452, 3465)	0.047	2079 (990, 4152)	1710 (231, 2520)	0.088
Age 22 (metabolic minutes/week)	2049 (933, 4158)	2307 (1074, 4236)	1416 (555, 3141)	0.045	2064 (933, 4158)	1902 (888, 3702)	0.746
Calories/day at age 14 (mean ± SD)	2078 ± 674	2044 ± 647	2155 ± 731	0.277	2065 ± 662	2145 ± 739	0.524
**Pregnancy/antenatal factors**							
Maternal antenatal history, *n* (%)							
Gestational diabetes	6 (2.64)	5 (3.11%)	1 (1.52%)	0.675	5 (2.63)	1 (2.70)	1.000
Pre-existing diabetes	5 (2.20)	4 (2.48%)	1 (1.52%)	1.000	4 (2.11)	1 (2.70)	0.593
Pre-eclampsia/eclampsia	45 (1.82)	34 (21.12%)	11 (16.67%)	0.445	38 (20.00)	7 (18.92)	0.880
Pre-term delivery < 37 weeks	20 (8.97)	14 (8.92%)	6 (9.09%)	0.967	15 (8.06)	5 (13.51)	0.289
Gestational age at delivery (mean ± SD)	39.15 ± 2.38	39.12 ± 2.28	39.23 ± 2.61	0.768	39.16 ± 2.26	39.14 ± 2.96	0.957

*t* test, Fisher’s exact test, Pearson *χ*^2^ test, and Mann-Whitney test were used for comparisons between the groups

*BMI* body mass index, *HA* hyperandrogenism, *IQR* interquartile range, *OA* oligo-anovulation, *PCOM* polycystic ovary morphology, *PCOS* polycystic ovary syndrome, *SD* standard deviation

### Cross-sectional BMI differences by diagnostic criteria

Table 3 details the participants’ mean BMI over time. Using the original Rotterdam criteria, participants with and without PCOS had similar BMI in childhood, but BMI in participants with PCOS was significantly greater than in those without PCOS from age 14 onwards (BMI at age 14, 22.8 ± 4.4 vs 21.0 ± 3.4, *p* = 0.003). Using the updated 2018 adolescent Rotterdam criteria, the divergence of BMI occurred earlier where participants with PCOS had higher BMI than participants without PCOS from age 5 onwards (BMI at age 5, 16.3 ± 2.0 kg/m^2^ vs 15.6 ± 1.4 kg/m^2^, *p* = 0.013).

**Table 3:** Cross-sectional comparison of BMI at each time point

	Age 1, *n* = 218	Age 2, *n* = 67	Age 3, *n* = 165	Age 5, *n* = 217	Age 8, *n* = 216	Age 10, *n* = 225	Age 14, *n* = 227	Age 16, *n* = 207	Age 20, *n* = 194	Age 22, *n* = 173
**Overall population (kg/m**^**2**^**)**										
	16.86 ± 1.33	15.79 ± 1.28	15.99 ± 1.20	15.69 ± 1.53	16.74 ± 2.09	18.32 ± 3.07	21.51 ± 3.81	23.08 ± 4.23	24.11 ± 4.77	25.03 ± 5.54
**Original Rotterdam criteria (kg/m**^**2**^**)**										
O-nPCOS	16.78 ± 1.33	15.67 ± 1.26	15.96 ± 1.21	15.62 ± 1.43	16.65 ± 1.97	18.15 ± 2.99	20.97 ± 3.41	22.58 ± 3.58	23.68 ± 4.28	24.24 ± 4.48
O-PCOS	17.04 ± 1.34	16.02 ± 1.33	16.07 ± 1.18	15.86 ± 1.76	16.96 ± 2.37	18.76 ± 3.24	22.82 ± 4.40	24.36 ± 5.37	25.40 ± 5.83	27.08 ± 7.31
*p* value	0.193	0.296	0.585	0.307	0.329	0.181	0.003	0.021	0.061	0.015
O-nPCOS (*n* = 161)	154	44	119	154	153	161	161	148	145	125
O-PCOS (*n* = 66)	64	23	46	63	63	64	66	59	49	48
**Updated 2018 adolescent Rotterdam criteria (kg/m**^**2**^**)**										
U-nPCOS	16.81 ± 1.36	15.72 ± 1.31	15.93 ± 1.22	15.58 ± 1.41	16.58 ± 1.92	18.13 ± 2.90	21.10 ± 2.99	22.54 ± 3.59	23.58 ± 4.20	24.19 ± 4.45
U-PCOS	17.09 ± 1.13	16.12 ± 1.14	16.34 ± 1.04	16.29 ± 2.00	17.58 ± 2.70	19.35 ± 3.76	24.19 ± 4.84	25.97 ± 5.96	27.56 ± 6.58	29.79 ± 8.22
*p* value	0.254	0.327	0.129	0.013	0.043	0.076	0.001	0.003	0.006	0.002
U-nPCOS (*n* = 190)	182	55	142	183	181	190	189	174	168	147
U-PCOS (*n* = 37)	36	12	23	34	35	35	37	33	28	26

*t* test was used for comparisons between the groups

*O-nPCOS* original non-polycystic ovary syndrome group, *O-PCOS* original Rotterdam polycystic ovary syndrome group, *U-nPCOS* updated non-polycystic ovary syndrome group, *U-PCOS* updated Rotterdam polycystic ovary syndrome group

### Longitudinal BMI change by diagnostic criteria and phenotypes

Table 4 shows the longitudinal models of BMI change over time stratified by PCOS status, adjusted for age of menarche, family income at age 14, smoking status at age 22, and marital status at age 22. Using the original Rotterdam criteria, participants with PCOS had significantly greater BMI increase than participants without PCOS from age 14 onwards (Wald test for overall differences *p* < 0.001). However, on the updated 2018 Rotterdam criteria, the BMI increase was greater in PCOS than in those without PCOS from age 10 onwards (Wald test for overall differences *p* < 0.001).

**Table 4:** Difference in longitudinal BMI change from baseline (age 1) stratified by PCOS diagnostic criteria

**Original Rotterdam criteria**							
	**Original** **non-PCOS** **(*n* = 161)**		**Original PCOS (*n* = 66)**		**Difference between the groups**		***p*** value*
	**∆BMI**	**95% CI**	**∆BMI**	**95% CI**	**∆BMI**	**95% CI**	
Age 1 (baseline)	–	–	–	–	–	–	
Age 2	− 1.31	− 2.14 to − 0.47	− 2.72	− 4.78 to − 0.65	− 1.47	− 3.32 to 0.37	0.117
Age 3	− 0.93	− 1.49 to − 0.37	− 1.19	− 2.63 to 0.25	− 0.27	− 1.53 to 1.00	0.680
Age 5	− 1.21	− 1.72 to − 0.69	− 1.35	− 2.68 to − 0.02	− 0.15	− 1.33 to 1.02	0.800
Age 8	− 0.18	− 0.70 to 0.34	− 0.25	− 1.57 to 1.07	− 0.08	− 1.25 to 1.09	0.895
Age 10	1.18	0.67 to 1.69	1.82	0.50 to 3.14	0.64	− 0.53 to 1.80	0.284
Age 14	4.24	3.73 to 4.75	5.83	4.52 to 7.14	1.59	0.43 to 2.75	0.007
Age 16	5.77	5.25 to 6.29	7.41	6.07 to 8.75	1.64	0.46 to 2.82	0.006
Age 20	6.85	6.33 to 7.37	7.94	6.59 to 9.29	1.10	− 0.09 to 2.29	0.070
Age 22	7.39	6.87 to 7.91	9.40	8.07 to 10.72	2.00	0.82 to 3.17	0.001
**Updated 2018 adolescent Rotterdam criteria**							
	**Updated** **non-PCOS** **(*n* = 190)**		**Updated PCOS (*n* = 37)**		**Difference between the groups**		***p*** value*
	**∆BMI**	**95% CI**	**∆BMI**	**95% CI**	∆BMI	95% CI	
Age 1 (baseline)	–	–	–	–	–	–	
Age 2	− 1.43	− 2.21 to − 0.65	− 2.57	− 5.63 to 0.49	− 1.27	− 3.43 to 0.89	0.248
Age 3	− 0.95	− 1.45 to − 0.44	− 1.86	− 4.38 to 0.67	− 0.96	− 2.68 to 0.77	0.278
Age 5	− 1.28	− 1.75 to − 0.80	− 1.02	− 3.21 to 1.17	0.23	− 1.28 to 1.73	0.770
Age 8	− 0.26	− 0.74 to 0.21	0.32	− 1.81 to 2.45	0.56	− 0.91 to 2.03	0.457
Age 10	1.14	0.67 to 1.61	2.73	0.57 to 4.89	1.55	0.07 to 3.04	0.041
Age 14	4.22	3.75 to 4.69	7.51	5.38 to 9.64	3.27	1.80 to 4.74	< 0.001
Age 16	5.70	5.22 to 6.17	9.60	7.41 to 11.79	3.86	2.35 to 5.37	< 0.001
Age 20	6.70	6.22 to 7.18	10.20	7.97 to 12.42	3.50	1.98 to 5.04	< 0.001
Age 22	7.27	6.79 to 7.74	12.17	10.01 to 14.33	4.89	3.40 to 6.39	< 0.001

Models were generated using generalized estimating equations and adjusted for age of menarche, family income at age 14, smoking status at age 22, and marital status at age 22

*p value for the interaction term PCOS status by age

*∆BMI* mean BMI change, *CI* confidence interval, *PCOS* polycystic ovary syndrome

**Fig. 1:**
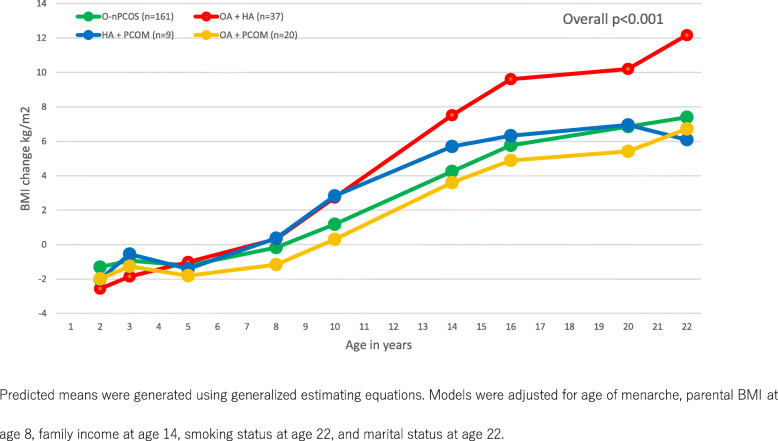
Predicted longitudinal BMI change over time by PCOS phenotypes

To examine if the updated 2018 Rotterdam criteria identified participants at risk of higher long-term BMI gain, we analyzed 3 PCOS phenotypes (updated Rotterdam criteria phenotype OA + HA and two phenotypes excluded by the updated criteria HA + PCOM and OA + PCOM), by time as an interaction term in the longitudinal analysis (Table 5). After adjusting for age of menarche, family income at age 14, smoking at age 22, and marital status at age 22, compared to those without PCOS, phenotype OA + HA (updated diagnostic criteria) had greater BMI increase from age 10, whilst phenotypes HA + PCOM and OA + PCOM had comparable BMI changes over time as participants without PCOS. The adjusted predicted mean change in BMI trajectories of each PCOS phenotype and participants without PCOS is shown in Fig. 1.

**Table 5:** Difference in longitudinal BMI change from baseline (age 1) stratified by PCOS phenotype

	Original non-PCOS (*n* = 161)		OA + HA (updated PCOS) (*n* = 37)			HA + PCOM (*n* = 9)			OA + PCOM (*n* = 20)		
	∆BMI	95% CI	∆BMI	95% CI	*p* value*	∆BMI	95% CI	*p* value^#^	∆BMI	95% CI	*p* value^$^
Age 1	–	–	–	–	–	–	–	–	–	–	–
Age 2	− 1.31	− 2.14 to − 0.47	− 2.57	− 5.63 to 0.49	0.200	− 2.02	− 4.66 to 0.61	0.745	− 1.99	− 4.94 to 0.95	0.692
Age 3	− 0.93	− 1.49 to − 0.37	− 1.86	− 4.38 to 0.67	0.273	− 0.55	− 2.83 to 1.73	0.849	− 1.27	− 2.65 to 0.10	0.694
Age 5	− 1.21	− 1.72 to − 0.69	− 1.02	− 3.21 to 1.17	0.844	− 1.41	− 3.35 to 0.54	0.888	− 1.81	− 3.18 to 0.43	0.487
Age 8	− 0.18	− 0.70 to 0.34	0.32	− 1.81 to 2.45	0.531	0.37	− 1.57 to 2.31	0.704	− 1.17	− 2.57 to 0.23	0.263
Age 10	1.18	0.67 to 1.69	2.73	0.57 to 4.89	0.047	2.82	0.88 to 4.77	0.253	0.31	− 1.07 to 1.68	0.314
Age 14	4.24	3.73 to 4.75	7.51	5.38 to 9.64	< 0.001	5.69	3.75 to 7.64	0.312	3.61	2.24 to 4.99	0.471
Age 16	5.77	5.25 to 6.29	9.60	7.41 to 11.79	< 0.001	6.33	4.39 to 8.28	0.696	4.89	3.49 to 6.29	0.322
Age 20	6.85	6.33 to 7.37	10.20	7.97 to 12.42	< 0.001	6.95	5.00 to 8.89	0.946	5.41	4.01 to 6.81	0.097
Age 22	7.39	6.87 to 7.91	12.17	10.01 to 14.33	< 0.001	6.09	4.14 to 8.03	0.365	6.74	5.32 to 8.13	0.436

Models were generated using generalized estimating equations and adjusted for age of menarche, family income at age 14, smoking status at age 22, and marital status at age 22

*∆BMI* mean BMI change, *CI* confidence interval, *HA* hyperandrogenism, *OA* oligo-anovulation, *PCOM* polycystic ovary morphology, *PCOS* polycystic ovary syndrome

*p value for the interaction term phenotype OA + HA by age, comparing OA + HA with original non-PCOS

^#^*p* value for the interaction term phenotype HA + PCOM by age, comparing HA + PCOM with original non-PCOS

^$^*p* value for the interaction term phenotype OA + PCOM by age, comparing OA + PCOM with original non-PCOS

## Discussion

To the best of our knowledge, the present study is the first community-based prospective cohort study of women with and without well-characterized PCOS diagnostic features assessed in adolescent years from birth until young adulthood. Our data clearly demonstrates that in the adolescent population, the 2018 international guideline updated Rotterdam criteria detected a lower prevalence of PCOS of 16.3% compared with 29.1% using the original Rotterdam criteria. The updated criteria also identified adolescents with PCOS with rapidly increasing BMI trajectory, with long-term weight gain known to increase PCOS severity. This study also provides novel insights into PCOS diagnostic phenotypes and important patterns of long-term weight gain by phenotype.

PCOS diagnosis in adolescents is controversial as the diagnostic features of OA, HA, and PCOM overlap with normal pubertal physiology [8]. The 2018 international PCOS guideline process involved comprehensive evidence synthesis and reached an evidence-informed consensus recommendation, while also highlighting evidence gaps and research priorities. Importantly, the guideline recommended that all PCOS diagnostic phenotypes should be captured in research to clarify the long-term natural history [8]. Longitudinal studies examining the natural history of PCOS are scarce, mainly limited to adults in clinical settings and to those with self-reported PCOS or BMI status [14–22]. An important and large population-based Northern Finland Birth Cohort study has reported that women with PCOS have earlier adiposity rebound (the second rise in BMI following a nadir in early childhood) (age 5.2 ± 1.0 vs 5.6 ± 0.9, *p* < 0.001) and that BMI trajectories deviate around this age [17]. However, the Finnish cohort included self-reported BMI, and self-reported irregular menstrual cycles and hirsutism, or PCOS status in adulthood [17]. The Finnish study was unable to explore key research priorities on the implications of the 2018 updated Rotterdam criteria including accurate BMI trajectories or the differential BMI patterns across diagnostic phenotypes [17].

The current study examines measured BMI trajectories in community-based adolescents with well-characterized PCOS features from birth until young adulthood. It shows that in adolescents, the prevalence of the original Rotterdam criteria including all phenotypes was 29.1%, and the prevalence using the updated Rotterdam criteria (HA and OA) was 16.3%, a prevalence similar to that seen in adults of 8–13% on systematic review and 12–18% in an Australian population using the Rotterdam criteria [1, 33]. Capturing 29% of adolescents under the original Rotterdam diagnostic criteria may contribute to over-diagnosis at this life stage, potentially causing unnecessary psychological distress and financial and treatment burden. The fear of over-diagnosis may also limit the willingness of clinicians to diagnose PCOS in adolescents despite clear evidence showing under-diagnosis and delayed diagnosis cause significant frustration in the PCOS community [4]. Our findings of an adolescent prevalence that is similar to that seen in adulthood when applying the updated criteria may be reassuring for both clinicians and adolescents affected by PCOS.

A key concern in excluding phenotypes HA and PCOM and OA and PCOM in the updated 2018 diagnostic criteria is uncertainty about their natural history and potential for preventable long-term adverse outcomes. Our study provides novel insights into the natural history of BMI trajectories across PCOS diagnostic criteria and phenotypes, with those adolescents meeting updated criteria (phenotype HA and OA) having the greatest BMI increase over time and the PCOM inclusive phenotypes having a similar BMI trajectory to those not affected by PCOS. Weight gain is the key contributor to PCOS severity and long-term reproductive, cardiometabolic, and psychological complications. Our data are consistent with those from adult women suggesting more severe metabolic features in women with the HA and OA phenotypes, potentially linked to the metabolic impact of HA [34]. Our study supports the international PCOS guideline-recommended updated Rotterdam diagnostic criteria in adolescents and suggests that the updated Rotterdam criteria will identify the group that clinicians need most to target for prevention with early lifestyle intervention. The mechanisms underpinning the divergence in BMI prepubertally noted in both the Finnish cohort and our data are unclear [17]. Other long-term implications including infertility, diabetes, and psychological implications may still be increased in adolescents with OA and PCOM and HA and PCOM phenotypes, with future studies in this cohort needed to address these gaps.

Strengths of our study include the prospective design and unselected community-based population, increasing generalizability. All anthropometric measurements were collected in a standardized manner which reduced measurement errors and recall bias. Multiple time points and low dropout rates increased statistical power. Most importantly, the PCOS phenotypes in our study population were well characterized. The present study also has limitations. The upper limit of HA was set at the top 25% of free testosterone in this study population due to the lack of standardized reference range in this age group and to accommodate the variation of the participants’ gynecological age. This cutoff range was also previously used in other published Raine Study papers [9, 24, 26, 28]. Given the lack of standardized adolescent definition of PCOM, our study used the latest consensus definition of PCOM for adults at the time (≥ 1 ovary ≥ 10cm^3^ in volume or ≥ 12 follicles between 2 and 9 mm diameter) which was based on a study performed with 7 MHz transvaginal ultrasound transducer [27, 35]. However, it is noteworthy that definitions of PCOM change over time with advances in ultrasound technology, and the latest 2018 PCOS guideline now recommends using a 8-MHz transducer and the threshold updated to ≥ 20 follicles between 2 and 9 mm diameter and/or an ovarian volume of ≥ 10 cm^3^. The assessment of PCOM was conducted via transabdominal pelvic ultrasound in our study because most of these girls were not yet sexually active. We recognize that transvaginal ultrasound is more accurate in measuring ovarian volume and antral follicle count; however, this is often inappropriate in adolescents [8]. Overall, 91% of our population were Caucasian, limiting generalizability to other ethnicities. The diagnostic PCOS features were evaluated in the adolescent population, and our findings do not aim to reflect diagnostic approaches in adulthood, where ultrasound and PCOM inclusion in the diagnostic criteria are recommended. Finally, we do not yet know whether the adolescent phenotype of PCOS persisted into adulthood and the prevalence of infertility, diabetes, and psychological health in this cohort.

## Conclusions

In conclusion, this study addresses key evidence gaps in PCOS literature and international research priorities, contributing novel findings on the reduced prevalence of PCOS using the updated Rotterdam diagnostic criteria in the adolescent population. We provide insight into the natural history of weight gain across PCOS diagnostic criteria and phenotypes in adolescents. We show that updated 2018 Rotterdam criteria requiring both HA and OA identify adolescents most at risk of excess weight gain as a key driver of PCOS severity, a group who should be targeted for early lifestyle intervention and prevention. Our findings support the 2018 international PCOS guideline’s updated Rotterdam diagnostic criteria and the omission of sonographic PCOM evaluation for adolescent PCOS diagnosis [8]. The use of the updated Rotterdam criteria may limit over-diagnosis of PCOS in adolescents, increase clinician confidence in accurate diagnosis in adolescents, and limit reciprocal under-diagnosis, currently rife in PCOS. Whilst the long-term natural history of clinical outcomes is yet to be elucidated, the 2018 international guideline recommends that adolescents who do not fulfill the updated Rotterdam criteria and have persistent oligo-anovulation or hyperandrogenism can be considered at risk for PCOS and be reassessed in adulthood.

## Data Availability

The data that support the findings of this study are available from the Raine Study, but restrictions apply to the availability of these data, which were used under license for the current study, and so are not publicly available. Data are however available from the authors upon reasonable request and with permission from the Raine Study.

## Abbreviations

AE-PCOS: Androgen Excess and Polycystic Ovary Syndrome Society
BMI: Body mass index
CI: Confidence interval
GEE: Generalized estimating equations
HA: Hyperandrogenism
IPAQ: International Physical Activity Questionnaire
IQR: Interquartile range
PCOM: Polycystic ovary morphology
PCOS: Polycystic ovary syndrome
OA: Oligo-anovulation
MET: Metabolic equivalent
NIH: National Institutes of Health
SD: Standard deviation
SHBG: Sex hormone-binding globulin
